# Usefulness of intraoperative insular electrocorticography in modified functional hemispherectomy

**DOI:** 10.1186/s12883-017-0940-0

**Published:** 2017-08-25

**Authors:** Gun-Ha Kim, Joo Hee Seo, James E. Baumgartner, Fatima Ajmal, Ki Hyeong Lee

**Affiliations:** 1 Department of Pediatrics, College of Medicine, Korea University, Seoul, South Korea; 2 Comprehensive Epilepsy Center, Florida Hospital for Children and Florida Hospital, 615 E. Rollins Street, Orlando, FL 32803 USA

**Keywords:** Insular cortex, Epilepsy, Epilepsy surgery, Seizure, Child, Pediatric

## Abstract

**Background:**

The insular cortex is not routinely removed in modified functional hemispherectomy due to the risk of injury to the main arteries and to deep structures. Our study evaluates the safety and usefulness of applying intraoperative electrocorticography (ECoG) on the insular during the hemispherectomy.

**Methods:**

We included all patients who underwent insular ECoG during a modified functional hemispherectomy from 2012 to 2015. After the surgery, the decision for further resection of the insular cortex was made based on the presence of electrographic seizures on ECoG.

**Results:**

The study included 19 patients (age, 6.4 ± 4.7 years, mean ± standard deviation). Electrographic seizures were identified in 5 patients (26.3%). Sixteen of the 19 patients (84.2%) became seizure-free with a follow-up duration of 3.1 ± 0.6 years and no vascular complication occurred.

**Conclusions:**

Intraoperative insular ECoG monitoring can be performed safely while providing a tailored approach for insular resection during modified hemispherectomy.

## Background

In the literature, the seizure outcome after hemispherectomy in children varies from 52 to 80% upon follow-up 1 year after surgery, and remains stable beyond 5 years at 58–63% [1–5]. The most common cause of surgical failure after hemispherectomy is incomplete disconnection [2]. Common areas of interest include the corpus callosum, frontal basal cortex, and insular cortex. With the use of intraoperative magnetic resonance imaging (MRI), we can complete the disconnection of the epileptic hemisphere to the remaining structures in the corpus callosum or frontal basal cortex with high reliability. Although there is a high possibility of insular involvement in intractable epilepsy suggesting hemispheric pathology, the insular cortex is not routinely removed, unless epileptogenic, due to the risk of injury to the main arteries on the surface of the insula and deep structures such as the basal ganglia. An insular seizure is not easily distinguishable from frontal- or temporal-onset seizures based on scalp electroencephalography (EEG) or clinical semiology [6–10]. Since techniques like MRI and positron emission tomography (PET) are not sensitive enough to examine the extent of the involvement of the insular cortex in the epileptogenic zone, stereoelectroencephalography or magnetoencephalography (MEG) are often used [9, 10].

This study aimed to evaluate the safety and usefulness of applying intraoperative insular electrocorticography (ECoG) in modified functional hemispherectomy.

## Methods

### Patients

We included all patients who underwent intraoperative ECoG monitoring on the insular cortex during modified functional hemispherectomy, at Florida Hospital for Children, from January 2012 to September 2015, and reviewed medical records retrospectively.

### Preoperative workup

Patients underwent detailed preoperative evaluation including prolonged video-EEG, 3-Tesla MRI (Siemens Healthcare, Erlangen, Germany), PET with F-18 fluorodeoxyglucose, ictal and interictal technetium 99 m single photon emission computed tomography with subsequent subtraction ictal SPECT co-registered with MRI, and neuropsychological evaluation. If required, MEG, functional MRI, and a Wada test were performed. The surgical decision for hemispherectomy was made based on the consensus of the epilepsy board meeting.

### Intraoperative procedures

Each patient underwent a modified functional hemispherectomy. After the hemispherectomy without insular resection, ECoG was recorded from frontal and temporal sides of the exposed insular cortex. We used an 8-contact strip electrodes for monitoring insular ECoG and monitored for 3–6 min on each side of insula. Total intravenous anaesthesia with Propofol and Fentanyl was used to minimize effect on cortical electrical activity during ECoG. No pharmacological activation was introduced during the recording. We removed the insular cortex if an electrographic seizure was recorded on the insular ECoG.

### Pathology results and outcome

Pathological findings were reported based on the consensus of International League Against Epilepsy (ILAE) diagnostic methods commission [11]. The seizure outcome was classified based on the ILAE classifications [12].

## Results

### Patient profile

**Fig. 1:**
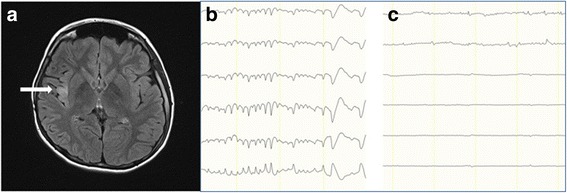
Insular hyperintensity shown on fluid-attenuated inversion-recovery MRI. 3-Tesla axial fluid-attenuated inversion-recovery images at the insular level show insular hyperintensity (**a**, arrow). The patient had an electrographic seizure on frontal strip of insular ECoG (**b**), which disappeared after insular resection (**c**)

A total of 19 patients were included in the current study (Table 1). Five patients (26.3%) had epilepsy surgery before hemispherectomy, three (15.8%) patients had an MRI suggestive of insular involvement (Fig. 1a), and four (21.1%) patients underwent intracranial EEG monitoring to confirm hemispheric involvement, since their MRIs did not clearly show hemispheric pathology (data not shown).

**Table 1:** Demographic data of patients with hemispherectomy

Total number of patients (*N*)	19
Sex, male/female (*N*)	8/11
Age at seizure onset, year, mean ± SD	1.2 ± 1.7
Age at surgery, year, mean ± SD	6.4 ± 4.7
Seizure duration, year, mean ± SD	5.32 ± 4.5
Number of seizures, per week, mean ± SD	28.0 ± 27.6
Epilepsy surgery prior to hemispherectomy (*N*)	
None	14
Lobectomy/topectomy ± corpus callosotomy	4
Corpus callosotomy only	1

*N* number of patients

### Intraoperative insular electrocorticography

In the entire cohort, electrographic seizures were identified in five patients (26.3%) by post-resection intraoperative ECoG on the insular cortex (Fig. 1b), of whom one patient had previous epileptic surgery, while another had brain tumor surgery after birth. Further, of the five patients, one had Rasmussen encephalitis, one had hemimegalencephaly, and three had diffuse malformation of cortical development (MCD). The characteristics of the patient with positive insular seizure are shown in Table 2.

**Table 2:** Characteristics of patients who had electrographic seizures on insular cortex during post-resection electrocorticography

Patient	Seizure		Past surgery	Scalp EEG		MRI	FDGPET	SPM PET	SISCOM
	Onset age, year	Frequency (per week)							
				Interictal	Ictal				
1	0.2	14	None	Lt/H	Lt/T, Lt/F	Hemi-megalencephaly	Lt/H	Lt/H	Lt/H
2	1.5	21	Rt/T lobectomy	Rt/F	Rt/H	Diffuse MCD	Rt/H	Rt/H	-
3	2.0	21	None	Rt/FC	Rt/FC	Diffuse MCD	Rt/H	Rt/H	-
4	5.0	70	None	Lt/H	Lt/H	Rasmussen encephalitis	Lt/H	-	-
5	0.1	70	Brain tumor resection after birth	Lt/H	Lt/H	Diffuse MCD	Lt/H	-	-

*EEG* electroencephalography, *FDG PET* 18 fluoro-2-deoxyglucose positron emission tomography scan, *MRI* magnetic resonance imaging, *SPM* statistical parametric mapping, *SISCOM* Subtraction ictal SPECT co-registered to MRI, *Lt* Left, *Rt* Right, *H* hemisphere, *F* frontal, *T* temporal, *FC* fronto-central, *MCD* malformations of cortical development, − not available

### Consistency between MRI and ECoG

As noted in Table 3, MRI was not always predictive of the presence of an insular seizure: MR fluid-attenuated inversion recovery (FLAIR) image showed no abnormality on insular cortex in 3 of 5 patients with an insular seizure, while MRI was abnormal on insular cortex in one patient without an insular seizure.

**Table 3:** Consistency between FLAIR MRI and intraoperative insular electrocorticography (N)

		High signal intensity on insular cortex on FLAIR image	
		Present	Absent
Insular seizure	Present	2	3
	Absent	1	13

*N* number of patients, *FLAIR* Fluid-attenuated inversion recovery

Presence of insular seizure according to etiology

Histopathological analyses revealed MCD as the most common etiology (10/19, 52.6%, Table 4), followed by perinatal stroke (6/19, 31.6%). Of them, insular seizures were identified on ECoG in 5 patients (26.3%) and pathology showed as follows: one with hemimegalencephaly, one with Rasmussen and three with diffuse malformations of cortical development. None of six perinatal stroke patients showed electrographic seizures on the insular ECoG. However, this difference was not statistically significant (*P* = 0.128).

**Table 4:** Presence of insular seizure according to pathology

Pathology	Total patients	Presence of Insular seizure on ECoG (N)	*p*-value
Developmental			0.128
Malformation of cortical development	10	3	
Hemimegalencephaly	1	1	
Inflammation			
Rasmussen encephalitis	2	1	
Vascular			
Perinatal stroke	6	0	

*N* number of patients, *ECoG* electrocorticography

Fisher’s exact test, statistical significance with *p* < 0.05

### Outcome

In the current study, 16 of the 19 patients (84.2%) became seizure free with a median follow-up of 3.1 ± 0.6 years (mean ± standard deviation) (Table 5). Four out of 5 patients (80%) with electrographic seizures on insular ECoG became seizure-free and one patient had breakthrough seizures with the onset from basal frontal brain area. Twelve of 14 patients (80%) without insular ECoG abnormality became seizure-free. Two patients with abnormality on both imaging and ECoG and one patient with an abnormal imaging but normal ECoG became seizure-free after surgery. Two patients developed hydrocephalus, and the disconnection was incomplete in the corpus callosum or basal frontal area in three patients. None of the patients developed perioperative stroke.

**Table 5:** Seizure outcome and surgical complication (total patients = 19)

Seizure-free	
Total	16/19 (84.2%)
Patients with insular seizure on ECoG	4/5 (80%)^a^
Complication	
Stroke	0
Infection	0
Hydrocephalus	2
Incomplete resection^b^	3

Data are number (%) or number unless otherwise stated. Mean follow-up duration of 3.1 (±0.6) years; ^a^ One patient had breakthrough seizures from basal frontal area of brain. ^b^ Incomplete resection on corpus callosum or basal frontal area, *ECoG* electrocorticography

## Discussion

In the current study, although electrographic seizures were detected on the insular ECoG in five patients (5/19; 26.3%), only two of these patients demonstrated subtle high signal intensity in the insular cortex on FLAIR images. These five patients underwent removal of the insular cortex in addition to functional hemispherectomy, and 84.2% of the patients in the current cohort were seizure-free with a mean follow-up duration of 3.1 years.

We believe the use of insular ECoG should be considered in all modified hemispherectomy cases for two reasons. Firstly, there is a relatively high possibility of insular involvement in hemispherectomy candidates. The existence of bidirectional interconnections between the amygdalo-hippocampal formation and the insula was confirmed by an electrophysiological study [13]. Given that the amygdalo-hippocampal formation is the most commonly involved brain structure in intractable epilepsy, the insular cortex should be considered the critical part of the epileptic network in these children. The residual insular cortex was positively correlated with persistent postoperative seizures in failed hemispherectomy patients [14].

Secondly, insular seizures could be indistinguishable from frontal or temporal lobe onset seizures [6–10]. The insular cortex is deeply located, buried in the lateral sulcus, and covered by the operculum, making it hard to detect using scalp EEG.

Although some previous research favored insular removal in hemispherectomy [15–17], others did not support it [2, 18]. Some centers routinely remove the insular cortex during hemispherectomy to prevent the potential development of persistent seizures [19, 20]. However, the routine removal of the insular cortex is not widely accepted at this point, due to the risk of injury to arteries and deep structures surrounding the insula (the average distance from the limen insulae to the putamen is only 5.7 mm [21]).

The current study also suggests the possible correlation between the pathology and the insular seizures on ECoG, although it did not reach the clinical significance. Except one patient with Rasmussen encephalitis, all 4 patients with electrographic seizures on insular ECoG had a developmental etiology; three had MCD and one had hemimegalencephaly. None of the five perinatal stroke patients showed insular seizure. These results suggest that developmental malformation commonly occurs in a more diffuse pattern, increasing the chances of insular cortex involvement. Due to the small number of patients in each pathology group, further research is required to validate the correlation between pathology and the involvement of the insular cortex in the epileptic network.

Our data support the use of insular ECoG as a safe and sensitive method to detect insular involvement in hemispherectomy patients. None of the 19 patients developed stroke or infection.

Limitations in the current study must be noted. The average duration of postoperative follow-up was less than 5 years. A recent multicenter study suggested that complications such as hydrocephalus could occur even after 8.5 years [22] and seizure outcome may change with a longer duration of follow-up [4]. In addition, intraoperative ECoG findings are not always predictive of postoperative seizure recurrence. Only a randomized controlled study could answer whether insular ECoG truly contributes to a better surgical outcome following hemispherectomy.

## Conclusions

Intraoperative insular ECoG monitoring could be performed safely without adding risk while providing a tailored approach to insular removal. Patients with developmental malformation may benefit from this approach.

## Abbreviations

ECoG: Electrocorticography
EEG: Electroencephalography
FLAIR: Fluid-attenuated inversion recovery
ILAE: International League Against Epilepsy
MCD: Malformations of cortical development
MRI: Magnetic resonance imaging
PET: Positron emission tomography
